# Selective CXCR4-Targeting Radioconjugates Derived from LY2510924: Evaluation of [^18^F]AlF-NOTA-SC, [^177^Lu]Lu-BL02, and [^161^Tb]Tb-BL02 for Theranostic Development

**DOI:** 10.3390/ph19081160

**Published:** 2026-07-25

**Authors:** Muriel Aline Spahn, Tom Van Loy, Christophe M. Deroose, Sofie Celen, Dominique Schols, Guy Bormans, Janke Kleynhans, Frederik Cleeren

**Affiliations:** 1 Radiopharmaceutical Research, Department of Pharmaceutical and Pharmacological Sciences, Katholieke Universiteit Leuven, 3000 Leuven, Belgium; murielaline.spahn@nus.edu.sg (M.A.S.);; 2 Molecular Structural and Translational Virology Research Group, Rega Institute for Medical Research, Department of Microbiology, Immunology and Transplantation, Katholieke Universiteit Leuven, 3000 Leuven, Belgium; tom.vanloy@kuleuven.be (T.V.L.); dominique.schols@kuleuven.be (D.S.); 3 Nuclear Medicine and Molecular Imaging, Department of Imaging and Pathology, Katholieke Universiteit Leuven, 3000 Leuven, Belgium; 4 Department of Nuclear Medicine, University Hospitals UZ Leuven, 3000 Leuven, Belgium; 5 Department of Nuclear Chemistry, Faculty of Nuclear Sciences and Physical Engineering, Czech Technical University in Prague, 11519 Prague, Czech Republic; kleynjan@cvut.cz

**Keywords:** positron emission tomography (PET), CXCR4, targeted radionuclide therapy (TRNT), terbium-161, auger emission, theranostics

## Abstract

**Background/Objectives**: The chemokine receptor CXCR4 plays a pivotal role in tumor progression, metastasis, and therapy resistance and is frequently overexpressed in hematologic malignancies, including multiple myeloma and lymphoma. This study investigates a CXCR4-targeted theranostic platform comprising a PET imaging agent and two therapeutic radioconjugates derived from the high-affinity CXCR4 antagonist LY2510924. **Methods**: The PET tracer [^18^F]AlF-NOTA-SC and therapeutic radioconjugates [^177^Lu]Lu-BL02 and [^161^Tb]Tb-BL02 were synthesized and evaluated. Radiolabeling efficiency, molar activity, and in vitro binding affinity were assessed. Specificity and uptake were evaluated in CXCR4-expressing U87.CD4.CXCR4 and MM.1S cells, with cytotoxic potential being analyzed via clonogenic survival assays. In vivo biodistribution and pharmacokinetics were evaluated in MM.1S xenograft mouse models, supported by longitudinal SPECT imaging. **Results**: All radioconjugates were obtained with high radiochemical purity (>98%). The constructs showed nanomolar affinity for human CXCR4; in vitro assays confirmed specific uptake in CXCR4-positive cells, and both therapeutic agents demonstrated dose-dependent cytotoxicity. In vivo, all compounds displayed comparable tumor uptake with low off-target accumulation. Co-injection studies confirmed consistent pharmacokinetics across agents, while SPECT/CT imaging demonstrated gradual tumor clearance of [^177^Lu]Lu-BL02 over seven days. **Conclusions**: The radiopharmaceutical trio [^18^F]AlF-NOTA-SC, [^177^Lu]Lu-BL02, and [^161^Tb]Tb-BL02 demonstrates the feasibility of a CXCR4-targeted theranostic approach for imaging and treating hematologic malignancies. The observed tumor washout highlights the need for further structural optimization to enhance tumor retention and therapeutic efficacy, providing a clear direction for future development toward clinical translation.

## 1. Introduction

The chemokine receptor CXCR4 is overexpressed across a range of malignancies, particularly hematologic cancers such as multiple myeloma and lymphoma. It plays a central role in tumor progression, metastasis, and therapy resistance by mediating tumor–stroma interactions and activating pro-survival signaling pathways [1]. Given its importance in cancer biology, CXCR4 has emerged as a highly attractive target for both diagnostic and therapeutic strategies. Targeting CXCR4 has also found its application in nuclear medicine [2], with [^68^Ga]Ga-PentixaFor, a cyclic peptide-based positron emission tomography (PET) radiopharmaceutical, being the most clinically advanced [3]. It demonstrates high specificity and excellent tumor-to-background contrast in various hematologic malignancies and often outperforms [^18^F]FDG by targeting CXCR4 receptor expression rather than glucose metabolism [4].

During the development of the therapeutic analogue PentixaTher, the chemical sensitivity of the peptide scaffold necessitated incorporation of an iodinated tyrosine residue in place of the non-functionalised tyrosine present in PentixaFor. As a result, Pentixather exhibits increased lipophilicity and increased plasma protein binding, leading to greater hepatobiliary accumulation and a biodistribution profile that differs substantially from that of PentixaFor. In addition, PentixaTher displays an approximately four-fold higher binding affinity than its diagnostic counterpart [5,6]. This discrepancy limits the theranostic compatibility of the pair and complicates accurate dosimetry based on the corresponding PET imaging. Consequently, there remains a need for alternative scaffold designs with more closely matched diagnostic and therapeutic properties.

**Figure 1:**
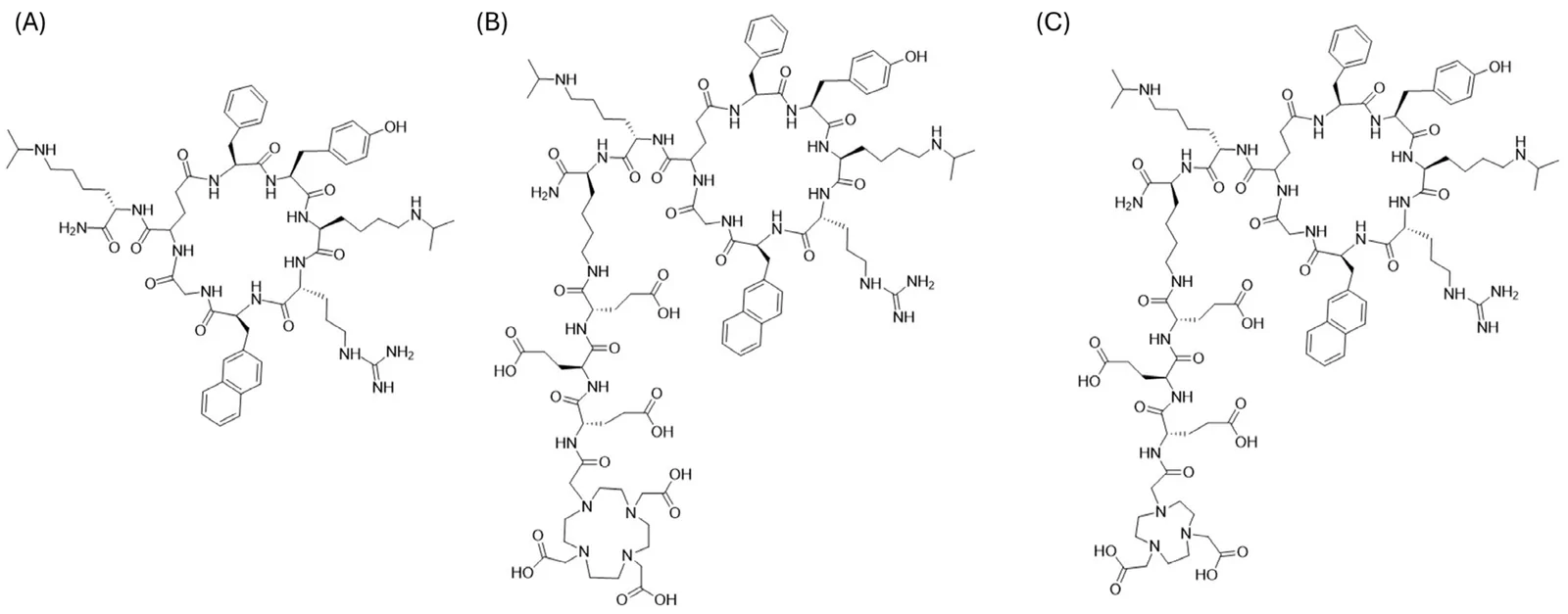
Chemical structure of (**A**) LY2510924, (**B**) DOTA-BL02 and (**C**) NOTA-SC.

One promising scaffold is LY2510924 (Figure 1A), a cyclic peptide (cyclo[Phe-Tyr-Lys(iPr)-D-Arg-2-Nal-Gly-D-Glu]-Lys(iPr)-NH_2_) engineered for high-affinity, antagonistic CXCR4 binding with enhanced in vivo stability through incorporation of D-amino acids and non-natural residues [7,8]. Building on this scaffold, Kwon et al. developed BL02, a cyclic pentapeptide derivative of LY2510924 modified with a DOTA chelator to enable radiometal labeling (Figure 1B) [9]. In mantle cell lymphoma models, [^68^Ga]Ga-BL02 provided high-contrast PET imaging with minimal off-target uptake, while [^177^Lu]Lu-BL02 therapy reduced tumor burden and extended survival, with matched pharmacokinetics confirming their suitability as a theranostic pair [9]. These findings support further clinical exploration of BL02-based radiopharmaceuticals in hematologic malignancies with high CXCR4 expression.

Given the logistical and production advantages of fluorine-18 over gallium-68, we previously developed an ^18^F-labeled PET tracer with pharmacokinetic properties closely matching those of BL02. The LY2510924-derived analogue NOTA-SC (Figure 1C) incorporates a triglutamate linker and a NOTA chelator at the C-terminus, enabling efficient fluorine-18 labeling via the Al^18^F-method [10]. In our earlier study, we demonstrated that [^18^F]AlF-NOTA-SC possesses highly favorable pharmacokinetic characteristics, including strong tumor uptake, low off-target accumulation, and superior imaging performance compared with [^18^F]FDG. Moreover, PET imaging in a non-human primate showed selective uptake in CXCR4-rich tissues, including the bone marrow and spleen, together with low hepatic accumulation and rapid renal clearance [10]. Collectively, these findings identified [^18^F]AlF-NOTA-SC as a promising CXCR4-targeted PET tracer with strong translational potential. Accordingly, GMP-compliant production is currently being implemented, and a first-in-human clinical trial at UZ Leuven is planned to commence beginning 2027.

The present study constitutes a critical step in the development of this theranostic platform. Here, we investigate the biodistribution and therapeutic efficacy of ^177^Lu- and ^161^Tb-labeled BL02 and evaluate the suitability of [^18^F]AlF-NOTA-SC as a matched diagnostic imaging partner. Demonstrating concordant in vivo behavior between the diagnostic and therapeutic counterparts is essential for enabling accurate patient selection, treatment planning, and dosimetry within a CXCR4-targeted theranostic framework.

This investigation compares [^177^Lu]Lu-BL02 and [^161^Tb]Tb-BL02 to determine whether the substantially higher emission of conversion and Auger electrons by terbium-161 (~12.12 electrons per decay, ~36.1 keV) relative to lutetium-177 (~1.11 electron per decay, ~1.0 keV) translates into enhanced cytotoxic efficacy, as previously reported [11,12], while preserving comparable tumor targeting and pharmacokinetic characteristics. Such properties may be particularly advantageous for the treatment of micrometastatic and disseminated hematological disease, were short-range, high-linear energy transfer electron emissions can increase localized energy deposition.

In the present study, we evaluated the potential of [^177^Lu]Lu-BL02 and [^161^Tb]Tb-BL02 as therapeutic counterparts to the diagnostic PET tracer [^18^F]AlF-NOTA-SC within a CXCR4-targeted theranostic framework. Following radiolabeling and purification, the in vitro CXCR4-binding affinity and effects on tumor cell survival were assessed. Subsequently, ex vivo biodistribution studies and longitudinal SPECT/CT imaging were performed in MM.1S xenograft mouse models over a 7-day period to characterize the in vivo pharmacokinetics and tumor-targeting properties.

## 2. Results

### 2.1. Radiolabeling of [^18^F]AlF-NOTA-SC, [^177^Lu]Lu-BL02 and [^161^Tb]Tb-BL02

[^177^Lu]Lu-BL02 and [^161^Tb]Tb-BL02 were manually labeled with radiochemical conversions (RCC) exceeding 99% for both tracers on iTLC. The apparent molar activity was 399.35 ± 5.43 GBq/μmol (*n* = 4) for [^177^Lu]Lu-BL02 and 371.98 ± 4.87 GBq/μmol (*n* = 3) for [^161^Tb]Tb-BL02, with excellent radiochemical purity (RCP) above 99% for both tracers after purification and formulation as determined by radioHPLC (Figure S1). Radiotracer identity was confirmed by analyzing the relative retention of the main peak in the radiochromatogram with the reference compound ([^nat^Lu]Lu-BL02 or [^nat^Tb]Tb-BL02) in the UV chromatogram. BL02 and its corresponding ^nat^Lu- and ^nat^Tb-complexes showed identical retention times (tR = 12.7 min), indicating unchanged chromatographic behaviour upon metal coordination. The in vitro stability of [^177^Lu]Lu-BL02 and [^161^Tb]Tb-BL02 demonstrated an intactness of over 99% over a 7-day period in both the formulation buffer at room temperature and the human serum at 37 °C. [^18^F]AlF-NOTA-SC was obtained with a radiochemical yield of 21.0 ± 7.1%, a radiochemical purity of >98%, and a molar activity of 16.4 ± 3.6 GBq/µmol.

### 2.2. In Vitro Assessments

**Figure 2:**
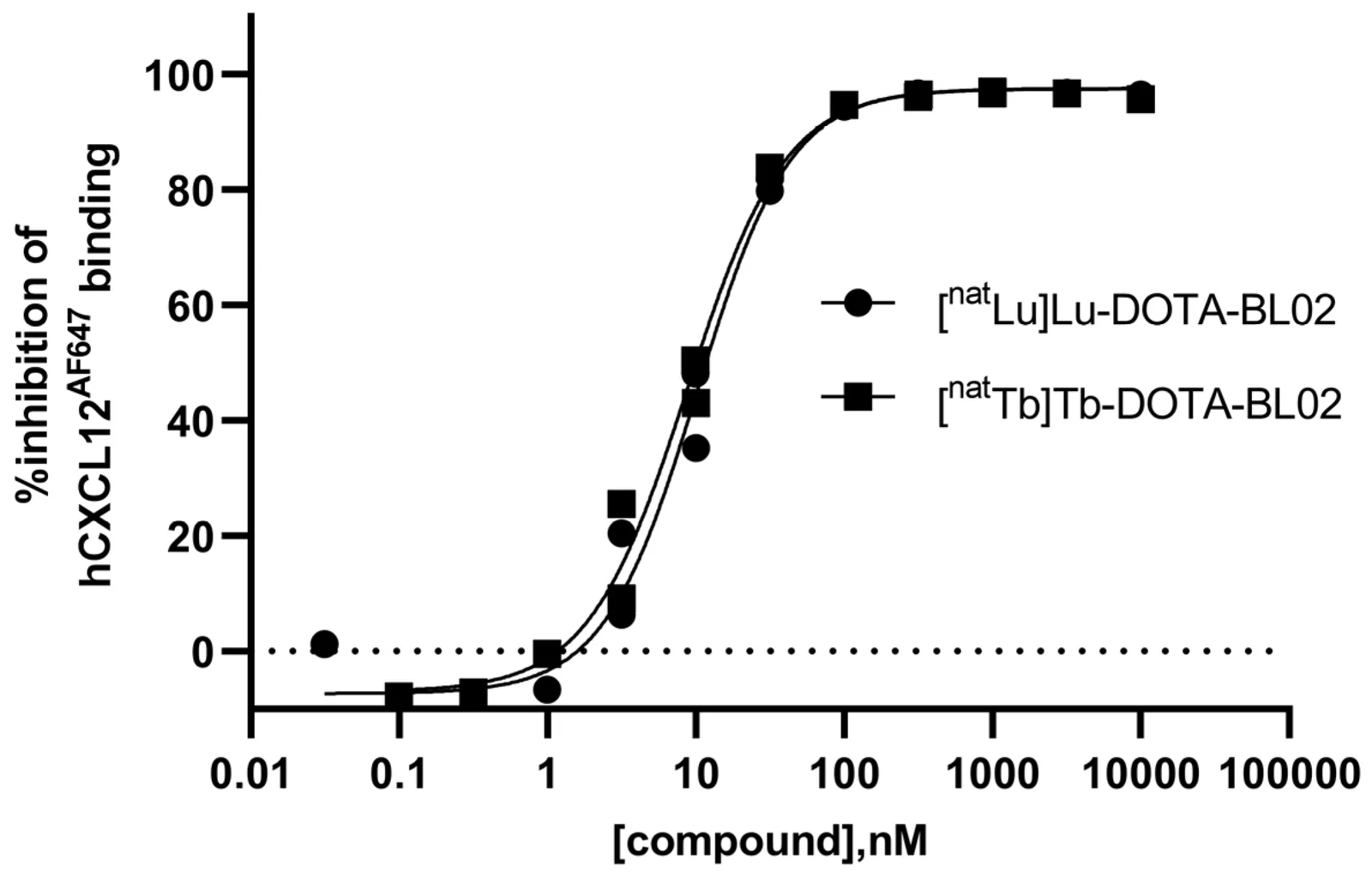
IC50 binding curve of [^nat^Lu]Lu-BL02 (●) and [^nat^Tb]Tb-BL02 (■) on human CXCR4. The values are expressed as mean ± SD (*n* = 4).

Both [^nat^Lu]Lu-BL02 and [^nat^Tb]Tb-BL02 demonstrated high affinity for human CXCR4, with IC50 values of 10.18 ± 2.01 nM and 10.84 ± 1.80 nM, respectively (Figure 2), comparable to the structurally related [^nat^F]AlF-NOTA-SC (IC_50_ 14.38 ± 2.65 nM). While their affinity was lower compared to the parent scaffold LY2510924 (IC_50_ = 0.36 ± 0.07 nM), it remained within the low-nanomolar range [8].

**Figure 3:**
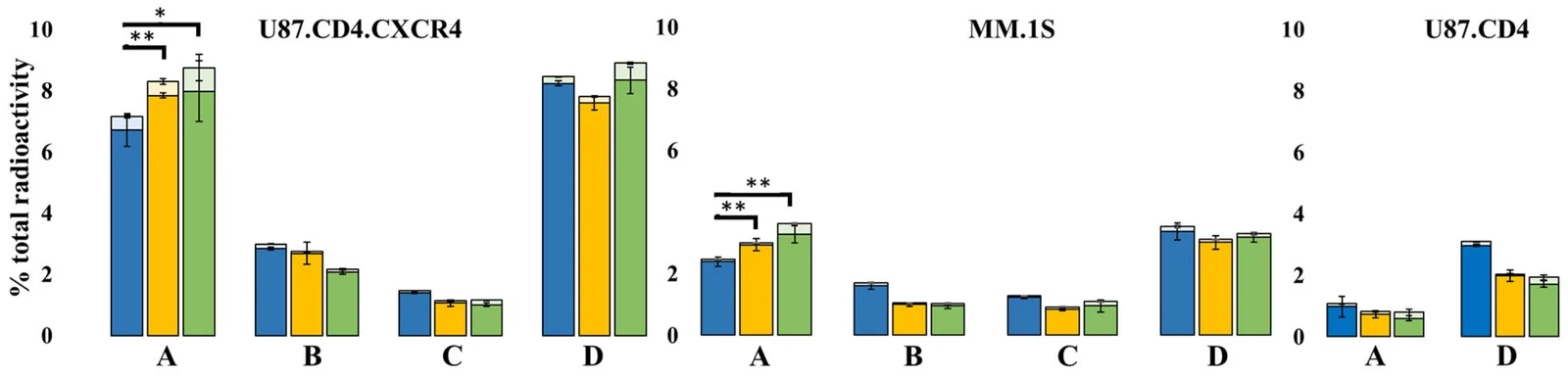
Radioligand binding assay of [^18^F]AlF-NOTA-SC [7] (blue), [^177^Lu]Lu-BL02 (yellow) and [^161^Tb]Tb-BL02 (green) in U87.CD4.CXCR4 and MM.1S cells. Bars represent total cellular uptake, with the upper segment indicating the internalized fraction and the lower segment the membrane-bound fraction, corrected to 0.2 × 10^6^ cells. Data are presented as mean ± SD (*n* = 3). Binding was assessed under four conditions: unblocked tracer only (A), co-incubation with AMD3100 (B), co-incubation with LY2510924 (C), and incubation at 4 °C (D). * *p* < 0.05, ** *p* < 0.001.

The U87.CD4.CXCR4 cell line was selected as a CXCR4-overexpressing model due to its stable and high receptor expression, enabling sensitive assessment of CXCR4-targeted binding, internalization, and radiobiological effects. In addition, MM.1S cells were included as a clinically relevant multiple myeloma model expressing endogenous CXCR4 to complement the overexpression system. Both [^177^Lu]Lu-BL02 and [^161^Tb]Tb-BL02 demonstrated significant uptake in CXCR4-overexpressing U87.CD4.CXCR4 cells, with total cellular uptake values of 8.29 ± 0.54% and 8.75 ± 1.76% of the applied activity, respectively (Figure 3). Of this, only 0.45 ± 0.10% ([^177^Lu]Lu-BL02) and 0.77 ± 0.44% ([^161^Tb]Tb-BL02) were internalized. Co-incubation with AMD3100 reduced total uptake to 2.74 ± 0.42% and 2.15 ± 0.15%, corresponding to reductions of 66.8% and 75.3%, respectively. Selfblocking with unlabeled LY2510924 further decreased uptake to 1.13 ± 0.18% and 1.15 ± 0.21%, reflecting reductions of 86.3% and 86.7%, respectively. Negligible uptake (<2%) was observed in cells lacking CXCR4 overexpression, confirming specificity (Figure 3). A similar uptake profile was observed in MM.1S cells. The total uptake of [^177^Lu]Lu-BL02 and [^161^Tb]Tb-BL02 was 2.99 ± 0.25% and 3.61 ± 0.62%, respectively. Co-incubation with AMD3100 resulted in a reduction in uptake by 64.7% and 71.5%, while co-incubation with LY2510924 reduced uptake by 69.2% and 69.9%, respectively (Figure 3). Incubation at 4 °C was performed to minimize energy-dependent cellular processes and to assess receptor binding in the absence of active internalization mechanisms. As expected for a CXCR4 antagonist, no significant internalization was observed under these conditions.

**Figure 4:**
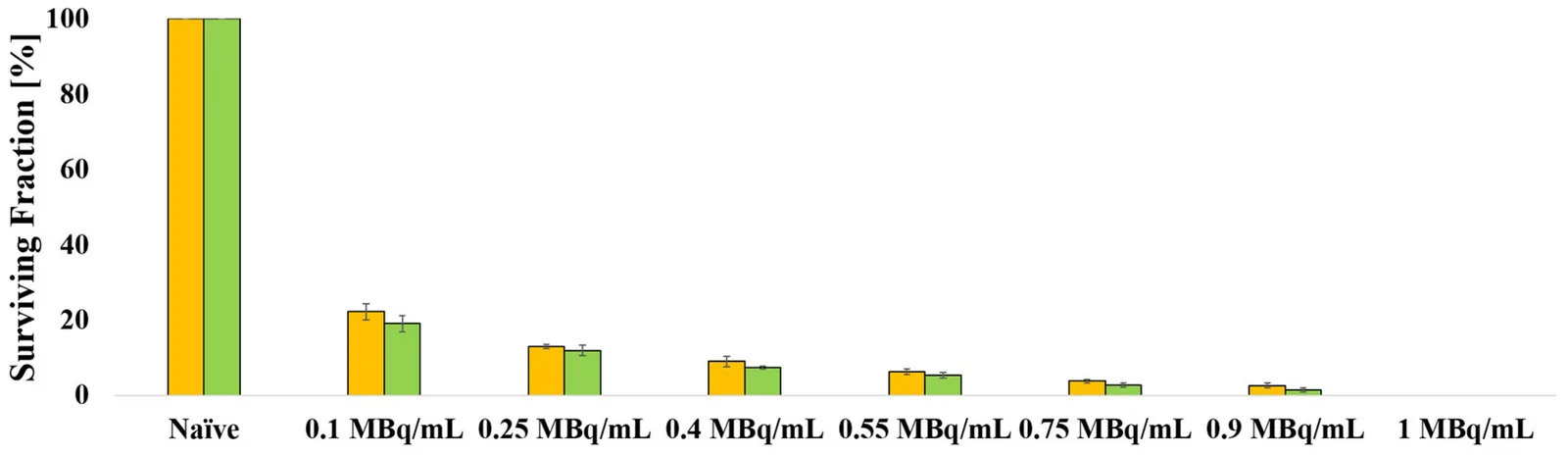
Clonogenic survival of U87.CD4.CXCR4 cells exposed to [^177^Lu]Lu-BL02 (yellow) or [^161^Tb]Tb-BL02 (green) (*n* = 3 per concentration).

Clonogenic assays were performed to assess the differences in cytotoxic effects but also reproductive survival of cells after exposure to [^177^Lu]Lu-BL02 or [^161^Tb]Tb-BL02. U87.CD4.CXCR4 cells were selected for clonogenic survival assays because their adherent growth characteristics allow reliable colony formation and quantitative evaluation of long-term reproductive survival. In contrast, MM.1S cells grow in suspension and do not consistently form colonies under conventional clonogenic assay conditions, precluding reliable clonogenic assessment. As shown in Figure 4, both radioconjugates induced a pronounced, activity-dependent decrease in surviving fraction relative to untreated controls. While [^161^Tb]Tb-BL02 consistently showed slightly lower survival fractions than [^177^Lu]Lu-BL02 across all tested activity levels (0.1–0.75 MBq/mL), these differences were not statistically significant.

### 2.3. In Vivo Studies

**Figure 5:**
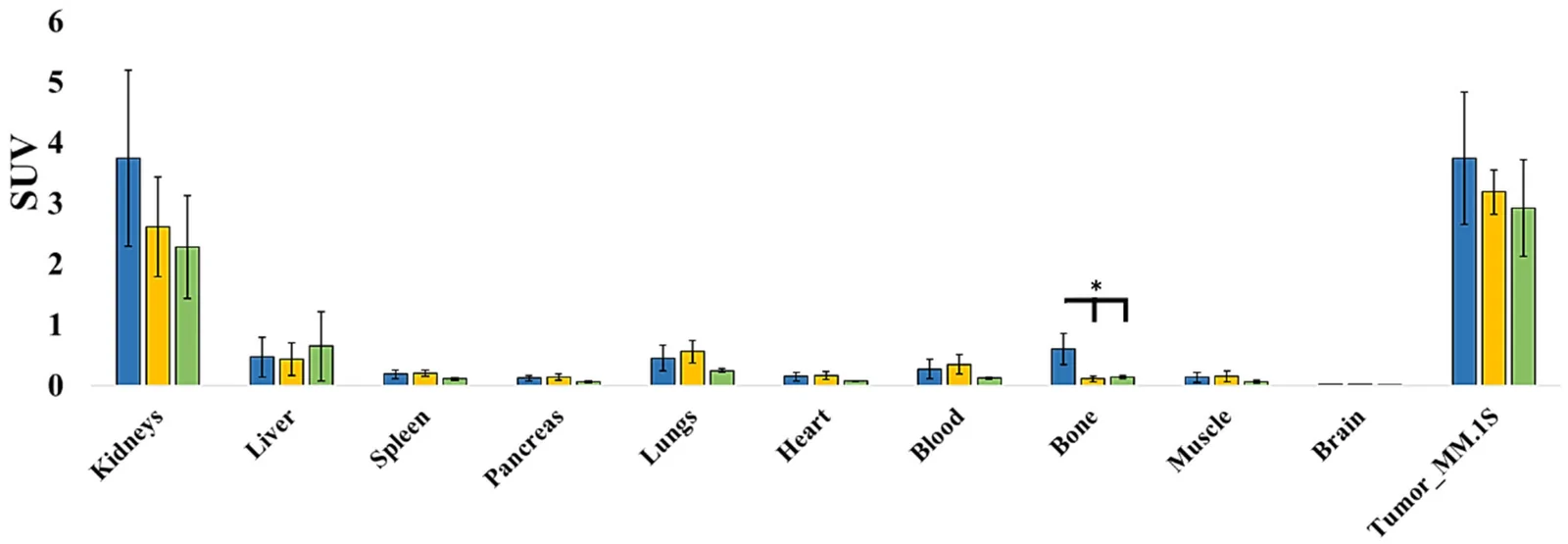
Ex vivo biodistribution profile of xenografted MM.1S mice co-injected with [^18^F]AlF-NOTA-SC (*n* = 8) (blue) and [^177^Lu]Lu-BL02 (*n* = 4) (yellow) or [^161^Tb]Tb-BL02 60 min *p*.i. (*n* = 4) (green). * *p* < 0.001.

The MM.1S xenograft model was selected for in vivo evaluation due to its endogenous CXCR4 expression and clinical relevance as a multiple myeloma model. In contrast to the CXCR4-overexpressing U87.CD4.CXCR4 cell line used for in vitro characterization, MM.1S cells provide a more clinically relevant setting to assess biodistribution and pharmacokinetic properties of CXCR4-targeted radiopharmaceuticals. At 60 min post-injection, biodistribution profiles were assessed in major organs and tumor tissues. All three radiopharmaceuticals displayed similar distribution patterns, with no statistically significant differences observed in most tissues, indicating well-matched pharmacokinetic behavior. Tumor uptake in the MM.1S xenografts was quantified as follows: [^18^F]AlF-NOTA-SC: 3.74 ± 1.09 SUV (*n* = 8), [^177^Lu]Lu-BL02: 3.19 ± 0.36 SUV (*n* = 4), and [^161^Tb]Tb-BL02: 2.93 ± 0.79 SUV (*n* = 4) (Figure 5, Tables S1–S3). These SUV values confirm comparable tumor accumulation among the three compounds.

A notable difference was observed in the skeleton, where [^18^F]AlF-NOTA-SC showed higher bone uptake (0.60 ± 0.25 SUV) compared to [^177^Lu]Lu-BL02 (0.11 ± 0.04 SUV) and [^161^Tb]Tb-BL02 (0.14 ± 0.02 SUV), consistent with minor in vivo defluorination of the ^18^F-labeled tracer.

**Figure 6:**
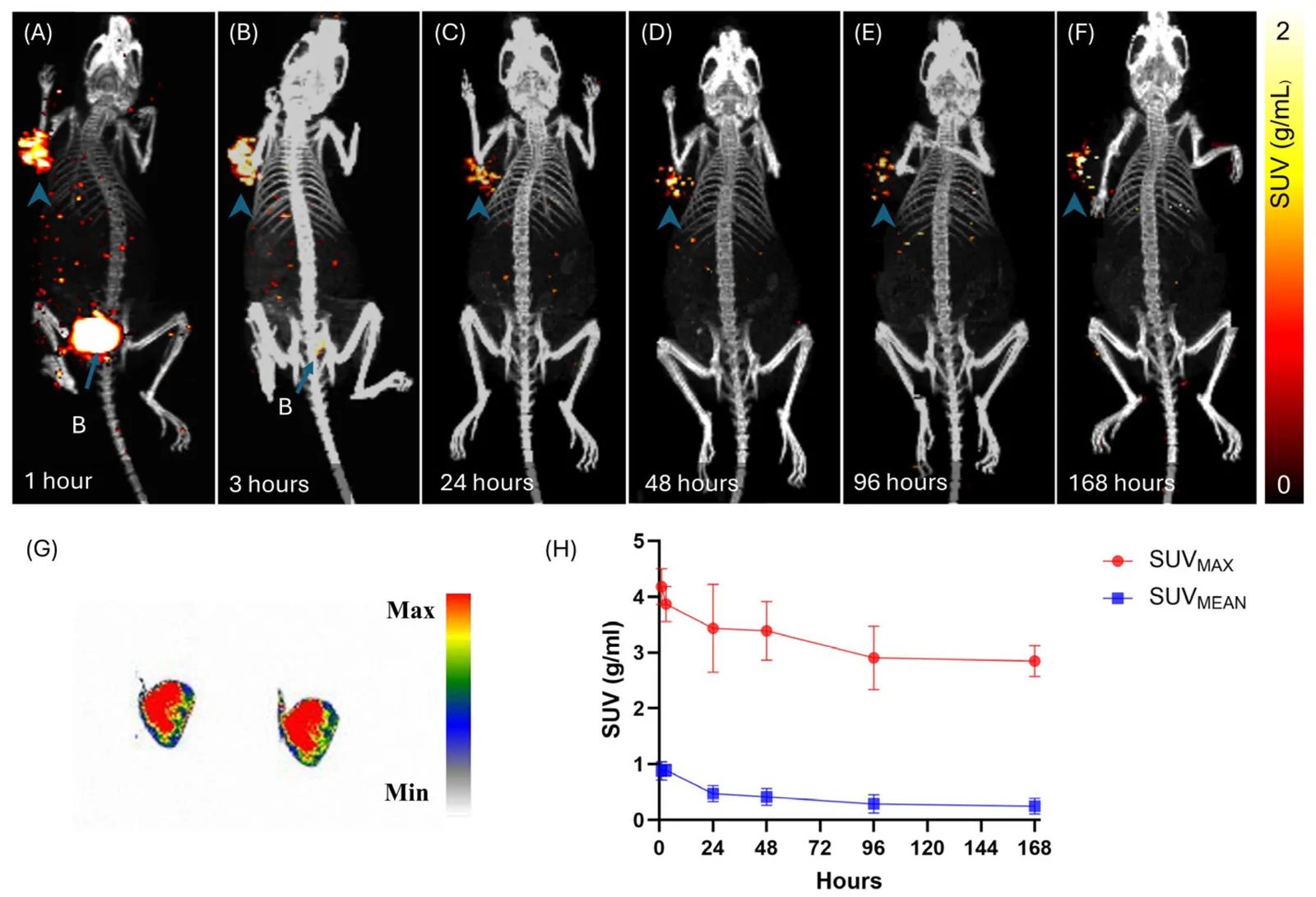
(**A**–**F**) Longitudinal maximum intensity projection SPECT/CT images of [^177^Lu]Lu-BL02 in MM.1S xenograft-bearing mice from 1 h to 7 days p.i., with tumor uptake indicated by the blue arrow, white B stands for bladder. (**G**) ex vivo autoradiography of tumor sections at 7d p.i. (**H**) Time activity curves showing SUVmax (red) and SUVmean (blue) as a function of time p.i.

To further characterize its pharmacokinetic profile and investigate the previously reported tumor washout of this CXCR4-targeted agent by Kwon et al. [9], longitudinal SPECT imaging was performed using the MM.1S tumor model. Consistent with the short-term biodistribution data (Figure 5), [^177^Lu]Lu-BL02 SPECT/CT demonstrated prominent accumulation in MM.1S tumors, which endogenously express CXCR4, while showing minimal uptake in healthy tissues (Figure 6A–F).

Tumor uptake of [^177^Lu]Lu-BL02 progressively declined over time; SUV_Max_ values started at 4.18 ± 0.262 g/mL at 1-h post-injection and declined to 2.84 ± 0.23 g/mL at 7 days post-injection. SUV_Mean_ values started at 0.88 ± 0.13 at 1 h post-injection and decline to 0.26 ± 0.11 g/mL at 7 days post-injection (Figure 6H). This gradual reduction highlights a significant therapeutic challenge, as reduced tumor retention may limit treatment efficacy and potentially contribute to tumor recurrence, progression, or metastasis. SPECT imaging results were in agreement with ex vivo biodistribution data at 7 days post-injection (Tables S4–S6). Autoradiographic analysis of tumor sections revealed a heterogeneous radiotracer distribution (Figure 6G), consistent with the observations reported [9].

## 3. Discussion

In comparison to previously published data on [^177^Lu]Lu-BL02, which reported an RCC of 67.5 ± 4.4% (*n* = 3) and an apparent molar activity of 247.65 ± 28.95 GBq/μmol (*n* = 3) [9], the radiolabeling method employed in this study achieved notably higher RCC and apparent molar activity. These enhancements are likely due to extended heating times and the use of a lower initial peptide amount, respectively. To the best of our knowledge, no comparable radiolabeling data are currently available for [^161^Tb]Tb-BL02.

Our affinity measurements differ from previous reports, which indicated no significant difference between LY2510924 (IC_50_ = 24.8 ± 2.5 nM) and [^nat^Lu]Lu-BL02 (IC_50_ = 22.7 ± 8.4 nM) [9]. Differences in apparent IC_50_ values between studies may arise from variations in experimental conditions, including cell line characteristics, CXCR4 expression levels, assay format, incubation conditions, tracer concentration, and the competing ligand system used. In particular, receptor density can influence the apparent affinity measured in competition binding assays, and therefore IC_50_ values should be interpreted within the context of the specific assay setup. A similar trend was observed for the structurally related [^nat^F]AlF-NOTA-SC (IC_50_ 14.38 ± 2.65 nM), which showed a reduced affinity compared to LY2510924 (IC_50_ 0.36 ± 0.07nM), when tested under our assay conditions. Despite these differences, the measured affinity of BL02 remains within the low nanomolar range and is comparable to that of [^nat^Ga]Ga-PentixaFor (IC_50_ 8.6 ± 1.1 nM) [13], supporting its potential for CXCR4-targeted applications.

The overall lower uptake observed in MM.1S cells, compared to U87.CD4.CXCR4 cells, is likely due to the lower CXCR4 expression levels in MM.1S, as previously confirmed by FACS analysis [10]. When compared to the imaging tracer [^18^F]AlF-NOTA-SC, both [^177^Lu]Lu-BL02 and [^161^Tb]Tb-BL02 exhibited similar binding profiles, yet both BL02 radioconjugates demonstrated significantly higher total cellular uptake in both U87.CD4.CXCR4 and MM.1S cells. This enhanced uptake is likely attributable to the slightly higher human CXCR4 binding affinity of BL02, as well as the increased molar activity of the radioconjugates, which may minimize competitive inhibition by non-radioactive peptides.

Clonogenic survival assays demonstrated that both therapeutic radioconjugates completely abrogated colony formation at activity concentrations ≥1 MBq/mL. Although the absolute differences in cytotoxicity between the two compounds were modest and did not reach statistical significance under the investigated conditions, [^161^Tb]Tb-BL02 showed a trend toward lower surviving fractions compared with [^177^Lu]Lu-BL02. This observation is consistent with the theoretical radiobiological advantages of ^161^Tb, including its higher linear energy transfer and additional emission of short-range Auger and internal conversion electrons, which may contribute to localized cellular damage. However, a potential therapeutic advantage of [^161^Tb]Tb-BL02 could not be conclusively demonstrated in the present study. These findings, together with the high cellular uptake observed in the binding assays, support the ability of both radioconjugates to efficiently target CXCR4-expressing cells and deliver cytotoxic radiation doses. Evaluation at lower activity concentrations and dedicated dosimetric studies may provide further insight into potential differences between [^177^Lu]Lu-BL02 and [^161^Tb]Tb-BL02, particularly under conditions where differences in radiation quality may become more apparent.

Biodistribution studies were conducted in immunocompromised SCID mice bearing subcutaneous MM.1S xenografts, a human multiple myeloma cell line that endogenously expresses CXCR4 and has previously been validated as a clinically relevant model for CXCR4-targeted imaging and therapy [10]. Moreover, MM.1S cells are known for their disseminated growth and bone marrow tropism in vivo, making them particularly well-suited for evaluating CXCR4-directed radiopharmaceuticals designed to treat bone marrow-infiltrating cancers [14].

To assess pharmacokinetic compatibility between the diagnostic and therapeutic agents, mice were co-injected intravenously with [^18^F]AlF-NOTA-SC and either [^177^Lu]Lu-BL02 or [^161^Tb]Tb-BL02. This co-injection strategy enabled a direct, side-by-side comparison of biodistribution under identical physiological conditions, thereby minimizing inter-animal variability and strengthening the reliability of organ and tumor uptake comparisons.

Importantly, SUVs of the diagnostic and therapeutic agents were largely consistent across tissues, falling within the same order of magnitude, which is a key requirement for effective theranostic pairing. The ability of [^18^F]AlF-NOTA-SC to reliably predict the tumor uptake of the therapeutic counterpart support its role in patient stratification and treatment planning.

While tumor uptake was well aligned between the radioconjugates, a discrepancy was observed in the skeleton, where [^18^F]AlF-NOTA-SC showed higher bone accumulation (0.60 ± 0.25 SUV) compared with [^177^Lu]Lu-BL02 (0.11 ± 0.04 SUV) and [^161^Tb]Tb-BL02 (0.14 ± 0.02 SUV). This finding likely reflects minor in vivo defluorination of the ^18^F-labeled compound, resulting in accumulation of free [^18^F]fluoride in bone. We have previously observed this phenomenon in preclinical studies, although it was not observed in the clinical setting with a different Al^18^F-labeled tracer [15]. In contrast, both therapeutic agents demonstrated excellent in vivo stability, as evidenced by minimal bone accumulation. Although skeletal background activity remained substantially lower than tumor uptake in the present study, the impact of potential defluorination-related bone signal on the detection of small marrow lesions will require further evaluation in clinical studies.

To further contextualize the findings, we compared our data to results from Kwon et al. [9], who evaluated [^177^Lu]Lu-BL02 in mantle cell lymphoma xenograft models (Z138 and GRANTA519) with differing CXCR4 expression levels. Although absolute tumor uptake values varied between models (ranging from ~3.5% to >17% ID/g at 1 h post-injection), the pharmacokinetic profiles including the temporal decline in tumor retention, were consistent with those observed in the MM.1S model. This suggests that the pharmacokinetic behavior of BL02-based tracers is largely preserved across CXCR4-expressing tumor types and that [^18^F]AlF-NOTA-SC can serve as a reliable surrogate for pre-therapeutic imaging. A clinical first-in-man trial is currently under preparation at UZ Leuven for the diagnostic application of [^18^F]AlF-NOTA-SC.

In summary, this co-injection biodistribution study at 1h p.i. provides strong evidence for the pharmacokinetic alignment of [^18^F]AlF-NOTA-SC with its therapeutic counterparts, [^177^Lu]Lu-BL02 and [^161^Tb]Tb-BL02. The comparable SUV values and overlapping organ distribution patterns support the use of this radiopharmaceutical trio as a matched theranostic platform for CXCR4-expressing hematologic malignancies, such as multiple myeloma.

Longitudinal SPECT imaging in our study revealed a progressive decline in tumor retention of [^177^Lu]Lu-BL02 over seven days. This pattern aligns with findings of Kwon et al. [9]. Using SPECT and ex vivo biodistribution analysis, they demonstrated tumor uptake that closely correlated with CXCR4 expression. In Z138 xenografts, uptake values were high (17.17 ± 3.04% ID/g) at 1h, decreasing to 3.57 ± 0.65% ID/g by 72h. GRANTA519 xenografts, which expressed lower CXCR4 levels, showed correspondingly lower uptake (6.83 ± 1.26% ID/g at 1h, 0.35 ± 0.03% ID/g at 72 h).

Notably, Z138 tumors also achieved exceptionally high tumor-to-background ratios. Tumor-to-blood ratios increased from 39.0 ± 3.0 to 630 ± 130 and tumor-to-muscle ratios rose from 167 ± 14 to 503 ± 127 between 1 and 4 h post-injection, remaining elevated at 24 h (873 ± 85 and 463 ± 90, respectively) and 72 h (673 ± 350 and 359 ± 59). While contrast in GRANTA519 models was less pronounced, tumor-to-blood ratios still increased markedly in the early time points. Furthermore, therapeutic studies by Kwon et al. [9] demonstrated significant tumor regression in Z138-bearing mice, underscoring the potential of [^177^Lu]Lu-BL02 despite its washout from tumor tissue.

Taken together, these findings highlight a consistent profile for [^177^Lu]Lu-BL02 with high initial tumor uptake followed by pronounced washout. Our MM.1S model mirrored this trend, suggesting that tumor washout is an intrinsic property of the compound rather than a model-specific phenomenon. The similarity across tumor types and expression profiles points to a structural-based limitation that warrants optimization. Given the highly comparable biodistribution profiles observed for [^177^Lu]Lu-BL02 and [^161^Tb]Tb-BL02, longitudinal SPECT imaging was performed only with [^177^Lu]Lu-BL02, as additional imaging with [^161^Tb]Tb-BL02 was not expected to provide substantially different pharmacokinetic information. Although CXCR4 specificity of BL02-based radioconjugates has previously been demonstrated, including for [^177^Lu]Lu-BL02 [9], future studies with optimized radioconjugates may include additional in vivo blocking experiments to further characterize receptor-mediated uptake in the tumor microenvironment.

Given the pharmacokinetic limitations, particularly the rapid tumor clearance, in vivo efficacy studies were not pursued at this stage. Before such studies can be meaningfully conducted, the pharmacokinetic profile of the compound must be optimized to ensure sufficient tumor retention, thereby maximizing the potential therapeutic benefit and enabling a reliable comparison between lutetium-177 and terbium-161.

To enhance tumor retention, several strategies are currently under active investigation. One promising approach involves multimerization of the targeting vector, which decreases the dissociation rate and extends tumor residence time. This strategy has been successfully demonstrated in other systems, such as FAPI dimers and multimers, which exhibited significantly improved tumor retention [16]. Another promising avenue is the use of covalent binding moieties, including sulfonyl fluoride and fluorosulfate groups, which irreversibly bind to target proteins. These have shown efficacy in enhancing retention in FAPI-based radiotracers. However, such modifications must be carefully controlled to avoid off-target interactions with endogenous nucleophilic residues [17].

Alternatively, aligning tracer kinetics with shorter-lived radionuclides offers a viable strategy. For instance, the alpha-emitting isotopes such as bismuth-213 (half-life: 45.6 min), astatine-211 (half-life: 7.2 h) or the in vivo alpha generator lead-212 (half-life: 10.64 h) possess high linear energy transfer and short tissue penetration ranges, enabling potent cytotoxic effects within a limited time window. These alpha-particles induce irreparable double-strand DNA breaks with minimal off-target damage, making them ideal for eradicating small tumor lesions, particularly in marrow-infiltrating malignancies such as multiple myeloma [18]. Future studies with optimized CXCR4-targeted radioconjugates should include comprehensive therapeutic evaluation, including assessment of potential off-target toxicity (e.g., renal toxicity) alongside therapeutic efficacy.

## 4. Materials and Methods

Chemicals and solvents were procured from Sigma-Aldrich (Bornem, Belgium), Fluka (Bornem, Belgium), Fisher (Doornik, Belgium), and Acros Organics (Geel, Belgium). All materials were of commercial grade and employed without further purification.

### 4.1. [^nat^Lu]Lu and [^nat^Tb]Tb Labelling of BL02

LY2510924 and BL02 were synthesized on demand by Pepmic Co., Ltd. (Suzhou, China). Non-radioactive BL02 complexes were prepared according to the procedure reported by Suzuki et al. [8]. Briefly, BL02 (300 µL, 2.3 mM in absolute ethanol; 1 equiv.; Mr = 2091.40 Da) was reacted with either LuCl_3_ or TbCl_3_ (220 µL, 23 mM in 0.4 M sodium acetate, pH 5; 10 equiv.) at 95 °C for 30 min. Following completion of the reaction and cooling to room temperature, the reaction mixture was diluted with 20 mL of HPCE water (Sigma-Aldrich) and purified using a preconditioned Sep-Pak Plus Light C18 cartridge (Waters, Eschborn, Germany), activated with 5 mL absolute ethanol followed by 5 mL water. The retained product was eluted with a mixture of 0.5 mL acetonitrile and 1 mL water, sterile-filtered through a Captiva PTFE + GF 0.45 µm syringe filter (Agilent Technologies, Santa Clara, CA, USA), rapidly frozen on dry ice, and lyophilized overnight.

Mass analysis of the construct was performed using a Dionex Ultimate 3000 UPLC system (Thermo Fisher Scientific, Sunnyvale, CA, USA) coupled sequentially to a UV detector and an ultra-high-resolution time-of-flight mass spectrometer (maXis Impact, Bruker Daltonics, Bremen, Germany) operating in positive electrospray ionization mode. Chromatographic separation was carried out on a Waters Acquity UPLC BEH C18 column (2.1 × 50 mm, 1.7 µm) maintained at 38.5 °C. Elution was performed at a flow rate of 0.6 mL/min using a gradient of Milli-Q water (solvent A) and acetonitrile (solvent B), both supplemented with 0.1% formic acid.

The multistep gradient consisted of the following steps: 0–2 min: 95% A; 2–8 min: from 95% → 5% A; 8–10 min: 5% A; and 10–12 min: 5% → 95% A. UV detection was performed at 220 nm throughout the chromatographic analysis. The average neutral molecular mass was determined using Compass Isotope Pattern software (version 3.2, Bruker Daltonics), while deconvolution of the acquired mass spectra was carried out with Compass DataAnalysis software (version 4.3, Bruker Daltonics). Expected 2263.34 Da ([^nat^Lu]Lu-BL02 C_99_H_144_N_23_O_27_Lu) and 2247.30 Da ([^nat^Tb]Tb-BL02 C_99_H_144_N_23_O_27_Tb); found 2263.02 Da ([^nat^Lu]Lu-BL02) and 2247.71 ([^nat^Tb]Tb-BL02).

### 4.2. Radiosynthesis of [^18^F]AlF-NOTA-SC, [^177^Lu]Lu-BL02 and [^161^Tb]Tb-BL02

The Al^18^F-labeling and quality control of [^18^F]AlF-NOTA-SC was performed as described earlier [10]. The radiochemical yield (RCY), radiochemical purity (RCP), and molar activity of the batch used in the present study are reported in the results section. [^177^Lu]LuCl3, non-carrier added, was purchased from ITM Isotopen Technologien Munchen AG, while [^161^Tb]TbCl3 from TerThera (Breda, The Netherlands). [^177^Lu]LuCl_3_ or [^161^Tb]TbCl_3_ (0.1–0.9 GBq) in 0.04 M HCl (10–100 μL) was added to a solution of the BL02 precursor (0.31 μg) in 300 µL of NaOAc buffer (0.1 M, pH 4.5).

Prior to radiolabeling, all buffers were treated with Chelex 100 resin (sodium form, 50–100 mesh; Sigma-Aldrich, Saint Louis, MO, USA) for 1 h to remove trace metal contaminants. Buffers and solvents were degassed and filtered before use. Radiolabeling was performed by incubating the reaction mixture at 100 °C for 25 min. Purification was achieved using a preconditioned Sep-Pak C18 Light cartridge (Waters, Eschborn, Germany), which was activated with 5 mL of absolute ethanol followed by 5 mL of water. After loading the crude reaction mixture, the cartridge was washed with 10 mL of water to remove uncomplexed radionuclides. The radiolabeled product was subsequently eluted with 0.25 mL of absolute ethanol into an Eppendorf tube and diluted to a final volume of 0.5 mL by adding 0.25 mL of 0.9% NaCl. To reduce the ethanol content, the vial was left open and placed on a thermoshaker at 60 °C and 2000 rpm for 15 min, allowing most of the ethanol to evaporate.

Radiochemical conversion (RCC) was determined by instant thin-layer chromatography (iTLC-SG; Varian, Diegem, Belgium) and radio-HPLC. For iTLC analysis, strips were developed in an elution chamber containing acetonitrile/water (75:25, *v*/*v*). Under these conditions, free [^177^Lu]LuCl_3_ and [^161^Tb]TbCl_3_ migrated with an *R*f value of approximately 0.2, whereas the corresponding radiolabeled complexes exhibited an *R*f of approximately 0.94. Activity distribution on the chromatograms was quantified by phosphor storage autoradiography (PerkinElmer, Waltham, MA, USA) using a Cyclone Plus imaging system and OptiQuant software (version 5.0, PerkinElmer).

Radiochemical purity (RCP) was further evaluated by radio-HPLC using a system consisting of an online degasser (Alltech Elite, Grace Davison Discovery Sciences, Lokeren, Belgium), an L-2130 pump (Hitachi, Tokyo, Japan), an L-2400 UV detector (Hitachi, Tokyo, Japan), and a 3-inch NaI(Tl)-based radioactivity detector (GABI Star module, Elysia-Raytest, Angleur, Belgium). Chromatographic separation was performed on a Waters XBridge^®^ C18 column (3.5 µm, 3.0 × 100 mm) using a gradient of Milli-Q water (solvent A) and acetonitrile (solvent B), each containing 0.1% trifluoroacetic acid, at a flow rate of 0.8 mL/min. The gradient steps over a 25-min period were 0–5 min, 95% A, 5–10 min 95% to 70% A, 10–15 min 70% to 60% A, 15–25 min 60% to 5% A. Apparent molar activity was determined by dividing the decay-corrected activity of the purified radioconjugate by the amount of BL02 quantified using an analytical HPLC calibration curve.

In vitro stability was assessed by RadioHPLC by incubating the radiotracer for 7 days at 37 °C in human serum (H4522 human male AB plasma, USA origin, sterile-filtered, Sigma Aldrich) and at room temperature in a formulation buffer composed of PBS supplemented with 0.58% sodium ascorbate.

### 4.3. In Vitro Assessment

#### 4.3.1. General Cell Culture Maintenance

The human glioblastoma cell line U87.CD4 was generously provided by Dr. D. Littman (The Skirball Institute of Biomolecular Medicine, New York, NY, USA) and served as the parental cell line for the generation of CXCR4-transfected cells, as previously described [19,20].

U87.CD4 cells were maintained in Dulbecco’s Modified Eagle Medium (DMEM) supplemented with 4 mM L-glutamine, 10% (*v*/*v*) fetal calf serum (Thermo Fisher Scientific, Waltham, MA, USA), 10 mM HEPES, and geneticin (0.2 mg/mL; Thermo Fisher Scientific). For maintenance of the CXCR4-transfected U87.CD4.CXCR4 cell line, the culture medium was additionally supplemented with puromycin (2 µg/mL; Sigma-Aldrich). Jurkat human T-cell leukemia cells expressing CXCR4 were obtained from the American Type Culture Collection (ATCC) and cultured in RPMI-1640 containing 10% fetal bovine serum (FBS) and 2 mM L-glutamine. MM.1S multiple myeloma cells (ATCC CRL-2974) were cultured in RPMI-1640 (Thermo Fisher Scientific) supplemented with 10% (*v*/*v*) fetal calf serum (Thermo Fisher Scientific). All cell lines were maintained in a humidified atmosphere at 37 °C with 5% CO_2_ and were routinely passaged twice weekly.

#### 4.3.2. Affinity Assessment and Radioligand Binding Assays

The affinity of the non-radioactive complexes for human CXCR4 was determined according to the method described by Schoofs et al. [21]. 100 µL of either [^nat^Lu]Lu-BL02 or [^nat^Tb]Tb-BL02 was dispensed into a 96-well plate as a serial dilution (0.15–10,000 nM) and combined with 100 µL assay buffer consisting of 20 mM HEPES (Thermo Fisher Scientific) and 0.2% BSA (Sigma-Aldrich) prepared in Hank’s Balanced Salt Solution (Thermo Fisher Scientific; pH 7.4). Subsequently, 50 µL containing 0.25 × 10^6^ Jurkat cells was added to each well, followed by 50 µL human CXCL12-AF647 (100 ng/mL; Almac, Craigavon, UK). Plates were incubated for 30 min at room temperature in the dark. After incubation, the cells were washed twice by centrifugation (400× *g*, 5 min) to replace the supernatant and subsequently fixed with 1% paraformaldehyde (Merck, Darmstadt, Germany). Fluorescence intensity was quantified by flow cytometry, and IC_50_ values were obtained by nonlinear regression analysis. All measurements were performed with at least four technical replicates.

Cell binding and internalization studies were carried out using 0.2 × 10^6^ U87.CD4, U87.CD4.CXCR4, or MM.1S cells seeded 24 h before the experiment. On the day of the assay, the culture medium was removed and approximately 150 kBq of the radiotracer, diluted in assay buffer (20 mM HEPES and 0.2% BSA in Hank’s Balanced Salt Solution, pH 7.4), was added to each well. For blocking experiments, a 75 µM concentration of the competitor was included simultaneously with the radiotracer, using either AMD3100 (Sigma-Aldrich) or LY2510924 (Pepmic) for homologous competition. Cells were incubated for 1 h at either 37 °C or 4 °C. Following incubation, the medium was collected after washing once with 600 µL of ice-cold PBS and designated as the “non-bound” fraction. Surface-bound activity was subsequently recovered by washing the cells twice for 5 min with 600 µL of ice-cold 50 mM glycine-HCl buffer (pH 2.8); these combined washes constituted the “membrane-bound” fraction. Intracellular activity was then determined after cell lysis with 500 µL of Solution A100 (ChemoMetec, Allerød, Denmark) and 500 µL of Reagent B (ChemoMetec), followed by an additional 500 µL PBS wash. These combined lysate fractions were defined as the “internalized” fraction. Radioactivity in all collected fractions was measured using a Wizard^2^ 2480 γ-counter (PerkinElmer, Waltham, MA, USA), while cell numbers were determined with a NucleoCounter^®^ NC-100™ (ChemoMetec). Membrane-bound and internalized activities were normalized to 0.2 × 10^6^ cells. All experiments were performed in triplicate.

For the suspension MM.1S cell line, the same protocol was followed with the exception of the washing steps. Instead of aspiration, cells were pelleted by centrifugation (400× *g*, 5 min) before removal of the supernatant after each wash to enable quantification of the non-bound, membrane-bound, and internalized fractions.

#### 4.3.3. In Vitro Clonogenic Assay

Clonogenic assays were carried out as described by Poty et al. [22]. A total of 3000 U87.CD4.CXCR4 cells were seeded into 6-well plates and allowed to adhere for 4 h at 37 °C in a humidified incubator with 2.5% CO_2_. Following incubation, cells were exposed to either [^177^Lu]Lu-BL02 or [^161^Tb]Tb-BL01 (ranging from 0.1 to 1.8 MBq/mL) or received a control treatment consisting of cell culture media with vehicle only. After two hours of exposure, the treatment medium was removed, and the cells were rinsed with PBS. Fresh growth medium (2.5 mL) was then added, and the cells were cultured for an additional 10 days under the same conditions. At the end of the incubation period, media was discarded and cells were gently washed with PBS before being fixed and stained using 3 mL of a solution containing 6% glutaraldehyde and 0.5% crystal violet. Colonies were counted manually, including those comprising at least 50 cells. Plating efficiency was calculated as the number of colonies formed in untreated wells divided by the number of cells originally seeded. The surviving fraction for each treatment condition was determined by dividing the number of colonies formed post-treatment by the number of cells plated, normalized to the plating efficiency.

### 4.4. In Vivo Assessment

Four-week-old female SCID mice (CB17.Cg-PrkdcscidLystbg-J/Crl, Charles River Laboratories, Sulzfeld, Germany) were included in the study and housed in individually ventilated cages under controlled environmental conditions with continuous temperature and humidity monitoring. Animals had unrestricted access to food and water, and were maintained on a 12 h light/12 h dark cycle. All animal procedures were reviewed and approved by the KU Leuven ethical review board (reference P010/2023) and performed in accordance with Directive 2010/63/EU [23].

The MM.1S xenograft model was generated following the procedure reported by Burke et al. [24]. Briefly, 1.5 × 10^6^ MM.1S cells were prepared in a 1:1 mixture with Cultrex Basement Membrane Extract (R&D Systems, Minneapolis, MN, USA) and subcutaneously injected into the shoulder region of four-week-old female SCID mice (CB17.Cg-PrkdcscidLystbg-J/Crl; Charles River Laboratories). Tumor growth was monitored three times weekly using a Vernier caliper, and tumor volumes were calculated according to the formula height × length × width. Once tumors reached a volume greater than 200 mm^3^, animals were randomly assigned to the different experimental groups for the subsequent biodistribution study.

#### 4.4.1. Dual Isotope Ex-Vivo Biodistribution

MM.1S tumor-bearing mice were anesthetized (2.5% isoflurane in O_2_ at 1 L/min flow rate). All mice received the PET tracer i.v., whilst half of the group was then co-inject with the lutetium-177 counterpart, and the other half, with the terbium-161 therapeutic counterpart. Injected activity was [^18^F]AlF-NOTA-SC (1.5–3.0 MBq, 0.18 nmol/mouse, *n* = 8) and [^177^Lu]Lu-BL02 (2.5–3.0 MBq, 7.51 pmol/mouse, *n* = 4) or [^161^Tb]Tb-BL02 (2.5–3.0 MBq, 8.17 pmol/mouse, *n* = 4). Throughout the imaging procedure, mice were maintained under inhalation anesthesia using 2.5% isoflurane in oxygen (flow rate: 1 L/min). Body temperature and respiratory rate were continuously monitored during the experiment. At 75 min post-injection (p.i.), animals were euthanized by administration of 5% isoflurane overdose followed by decapitation. Blood samples and major organs were collected, after which tissues were weighed. Radioactivity levels in blood, organs, and remaining body fractions were quantified using an automated γ-counter (PerkinElmer 1480 Wizard 3q) equipped with a 3-inch NaI(Tl) well-type detector coupled to a multichannel analyzer and sample changer. Recorded counts were corrected for background signal, physical decay, and detector dead time. Samples were first measured for fluorine-18 on the day of the experiment using an appropriate energy window. Following the decay of fluorine-18 the same samples were remeasured 2 days later using radionuclide-specific energy windows for lutetium-177 or terbium-161, respectively. The values have been expressed in standardized uptake values ((SUV; tissue activity concentration (MBq/g)/[injected activity (MBq)/body weight (g)])).

#### 4.4.2. SPECT/CT Imaging and Analysis

MM.1S tumor-bearing mice were anesthetized with 2.5% isoflurane in oxygen (flow rate: 1 L/min) and intravenously administered [^177^Lu]Lu-BL02 (22.7–26.1 MBq; 56.8–65.3 pmol/mouse; *n* = 3). Animals remained under inhalation anesthesia during tracer administration and throughout the imaging procedures. Physiological parameters were continuously monitored during both SPECT and CT acquisitions.

Imaging was initiated with a scout scan using the X-CUBE system (Molecubes, Ghent, Belgium) with the animals positioned head-first in prone orientation to define the appropriate field of view (FOV). A CT acquisition was subsequently performed using the selected FOV, consisting of a single projection acquired with a 1 s exposure time and an X-ray tube voltage of 55 kVp. The total CT acquisition duration was 5 min. Following CT completion, the imaging bed was transferred to the γ-CUBE system (Molecubes) for static SPECT acquisition over 30 min. In vivo μSPECT/CT imaging was carried out at 1 h, 3 h, 24 h, 48 h, 96 h and 168 h post-injection. After the last timepoint all the animals were euthanized by decapitation.

SPECT images were reconstructed through a maximum-likelihood expectation-maximization (MLEM) algorithm using 50 iterations (Molecubes). A predetermined q-factor (149.33) was applied during postprocessing to enable the generation of SUV-scaled images by converting counts/cc to activity/cc. SPECT/CT fusion and analysis were performed with PFUS version 4.0 (PMOD Technologies, Fällanden, Switzerland). To calculate radiopharmaceutical accumulation in the tumors as SUV, the tumors were delineated manually based on the CT images.

### 4.5. Statistical Analysis

Results are expressed as the mean ± standard deviation (SD). Differences were considered statistically significant when *p* < 0.05, with significance determined using an independent two-sample t-test in Microsoft Excel.

## 5. Conclusions

In this study, we systematically evaluated [^177^Lu]Lu-BL02 and [^161^Tb]Tb-BL02 as CXCR4-targeted therapeutic radioligands and compared their biological performance with the PET imaging counterpart [^18^F]AlF-NOTA-SC. Both therapeutic radioligands were synthesized with excellent radiochemical purity and high molar activity and demonstrated high binding affinity toward human CXCR4 in vitro. Radioligand binding assays confirmed specific, CXCR4-mediated uptake in two distinct cell lines, while clonogenic assays demonstrated effective dose-dependent cytotoxicity for both compounds.

In vivo biodistribution in MM.1S xenograft models showed comparable tumor uptake and pharmacokinetic profiles among all three compounds, supporting the potential of [^18^F]AlF-NOTA-SC as a predictive imaging surrogate for therapy planning and response monitoring. Nevertheless, SPECT imaging revealed gradual tumor clearance of [^177^Lu]Lu-BL02 over seven days, indicating that further structural optimization may be required to improve tumor retention and maximize therapeutic efficacy.

## Data Availability

The original contributions presented in this study are included in the article/Supplementary Materials. Further inquiries can be directed at the corresponding author.

## Supplementary Materials

The following supporting information can be downloaded at: https://www.mdpi.com/article/10.3390/ph19081160/s1, Figure S1 Chromatographic analysis of BL02, [^177^Lu]Lu-BL02 and [^161^Tb]Tb-BL02. (A) UV (220 nm) chromatogram of BL02 and radiochromatogram of [177Lu]Lu-BL02 (B) and [^161^Tb]Tb-BL02 (C). Biodistribution data (Tables S1–S6). Biodistribution data in MM.1S tumor-bearing mice were obtained at 1 h p.i. for [^18^F]AlF-NOTA-SC (*n* = 8), [^177^Lu]Lu-BL02 (*n* = 4), and [^161^Tb]Tb-BL02 (*n* = 4), and at 7 d p.i. for [^177^Lu]Lu-BL02 (*n* = 4).

## Abbreviations

The following abbreviations are used in this manuscript:

Al^18^F	Aluminum-[^18^F]fluoride
BSA	Bovine Serum Albumin
CT	Computed Tomography
CXCR4	C-X-C Chemokine Receptor Type 4
DMEM	Dulbecco’s Modified Eagle Medium
DNA	Deoxyribonucleic Acid
DOTA	1,4,7,10-Tetraazacyclododecane-1,4,7,10-Tetraacetic Acid
FACS	Fluorescence-Activated Cell Sorting
FAPI	Fibroblast Activation Protein Inhibitor
FBS	Fetal Bovin Serum
[^18^F]FDG	[^18^F]Fluorodeoxyglucose
GBq	Gigabecquerel
GMP	Good Manufacturing Practice
HEPES	4-(2-Hydroxyethyl)-1-piperazineethanesulfonic Acid
HPLC	High-Performance Liquid Chromatography
IC_50_	Half-Maximal Inhibitory Concentration
ID/g	Percentage injected dose per gram of tissue
iTLC	Instant Thin-Layer Chromatography
MBq	Megabecquerel
NOTA	Multiple Myeloma
PBS	Phosphate-Buffered Saline
PET	Positron Emission Tomography
RCC	Radiochemical Conversion
RCP	Radiochemical Purity
rF	Retention Factor/Retardation Factor
RPMI	Roswell Park Memorial Institute Medium
SCID	Severe Combined Immunodeficiency
SPECT	Single-Photon Emission Computed Tomography
SUV	Standardized Uptake Value
SUV_MEAN_	Mean Standardized Uptake Value
SUV_MAX_	Maximum Standardized Uptake Value
TRNT	Targeted Radionuclide Therapy
UPLC	Ultra-Performance Liquid Chromatography
UV	Ultraviolet
