# Supplementary material for: Selective CXCR4-Targeting Radioconjugates Derived from LY2510924: Evaluation of [18F]AlF-NOTA-SC, [177Lu]Lu-BL02, and [161Tb]Tb-BL02 for Theranostic Development
Source: Pharmaceuticals (Basel). 2026 Jul 25;19(8):1160. doi: 10.3390/ph19081160 (PMC13516207; doi:10.3390/ph19081160)
Supplement: Supplementary file 1 [file pharmaceuticals-19-01160-s001.zip › pharmaceuticals-4382476-supplementary.pdf]

pharmaceuticals

Supplementary data to:

Selective CXCR4-Targeting Radioconjugates Derived from LY2510924: Evaluation of [^{18}F]AlF-NOTA-SC, [^{177}Lu]Lu-BL02, and [^{161}Tb]Tb-BL02 for Theranostic Development

Muriel Aline Spahn [1], Tom Van Loy [2], Christophe M. Deroose [3,4], Sofie Celen [4], Dominique Schols [2], Guy Bormans [1], Janke Kleynhans [5] and Frederik Cleeren [2,*]

1 Radiopharmaceutical Research, Department of Pharmaceutical and Pharmacological Sciences, Katholieke Universiteit Leuven, 3000 Leuven, Belgium; murielaline.spahn@nus.edu.sg (M.A.S.)
2 Molecular Structural and Translational Virology Research Group, Rega Institute for Medical Research, Department of Microbiology, Immunology and Transplantation, Katholieke Universiteit Leuven, 3000 Leuven, Belgium; tom.vanloy@kuleuven.be (T.V.L.); dominique.schols@kuleuven.be (D.S.)
3 Nuclear Medicine and Molecular Imaging, Department of Imaging and Pathology, Katholieke Universiteit Leuven, 3000 Leuven, Belgium
4 Department of Nuclear Medicine, University Hospitals UZ Leuven, 3000 Leuven, Belgium
5 Department of Nuclear Chemistry, Faculty of Nuclear Sciences and Physical Engineering, Czech Technical University in Prague, 11519 Prague, Czech Republic; kleynjan@cvut.cz
* Correspondence: frederik.cleeren@kuleuven.be

Figure S1 Chromatographic analysis of BL02, [^{177}Lu]Lu-BL02 and [^{161}Tb]Tb-BL02. (A) UV (220 nm) chromatogram of BL02 and radiochromatogram of [^{177}Lu]Lu-BL02 (B) and [^{161}Tb]Tb-BL02 (C).

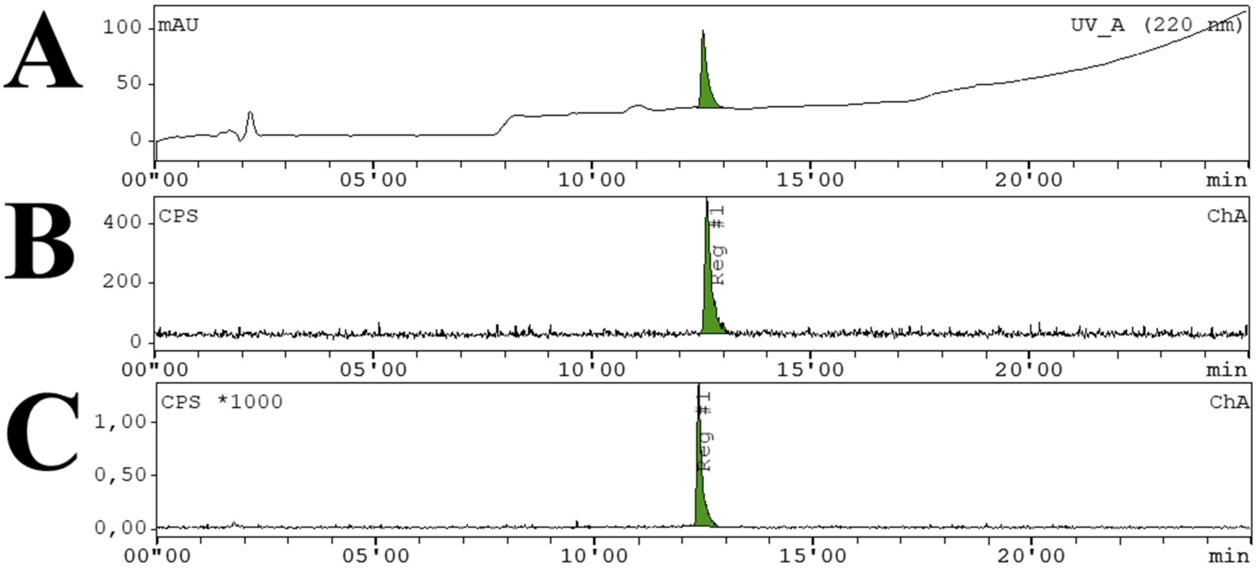

Biodistribution data

Biodistribution data in MM.1S tumor-bearing mice were obtained at 1 h p.i. for [^{18}F]AlF-NOTA-SC (n = 8), [^{177}Lu]Lu-BL02 (n = 4), and [^{161}Tb]Tb-BL02 (n = 4), and at 7 d p.i. for [^{177}Lu]Lu-BL02 (n = 4).

Table S1 SUV values of dissected organs from the *ex vivo* biodistribution study at 1 h p.i. injected with [^{18}F]AlF-NOTA-SC (n=8), [^{177}Lu]Lu-BL02 (n=4) and [^{161}Tb]Tb-BL02 (n=4)

Organ	[^{18}F]AlF-NOTA-SC	[^{177}Lu]Lu-BL02	[^{161}Tb]Tb-BL02
Kidneys	3.74±1.44	2.61±0.82	2.28±0.84
Liver	0.47±0.32	0.43±0.27	0.65±0.56
Spleen	0.19±0.07	0.20±0.05	0.11±0.01
Pancreas	0.12±0.04	0.14±0.05	0.06±0.01
Lungs	0.45±0.21	0.56±0.18	0.24±0.03
Heart	0.15±0.07	0.17±0.06	0.07±0.00
Blood	0.27±0.16	0.35±0.15	0.12±0.01
Bone	0.60±0.25	0.11±0.04	0.14±0.02
Muscle	0.14±0.08	0.15±0.08	0.06±0.02
Brain	0.02±0.01	0.02±0.01	0.00±0.00
Tumor_MM.1S	3.74±1.09	3.19±0.36	2.93±0.79

Table S2 Percent injected dose (%ID) values of dissected organs from the *ex vivo* biodistribution study at 1 h p.i. injected with [^{18}F]AlF-NOTA-SC (n=8), [^{177}Lu]Lu-BL02 (n=4) and [^{161}Tb]Tb-BL02 (n=4)

Organ	[^{18}F]AlF-NOTA-SC	[^{177}Lu]Lu-BL02	[^{161}Tb]Tb-BL02
Kidneys	5.44±1.99	4.20±1.35	2.64±0.99
Liver	1.69±0.53	1.64±0.33	1.98±0.23
Spleen	0.10±0.04	0.10±0.03	0.06±0.00
Pancreas	0.12±0.04	0.13±0.05	0.07±0.02
Lungs	0.38±0.20	0.49±0.18	0.19±0.00
Heart	0.07±0.03	0.09±0.03	0.03±0.00
Blood	1.91±1.12	2.46±1.11	0.89±0.12
Bone	7.13±2.98	1.33±0.45	1.68±0.33
Muscle	5.52±3.25	6.18±3.26	2.57±1.12
Brain	0.03±0.02	0.04±0.01	0.01±0.00
Tumor_MM.1S	15.26±10.40	6.93±2.25	13.37±3.81

Table S3 Percent injected dose per gram of tissue (%ID/g) values of dissected organs from the *ex vivo* biodistribution study at 1 h p.i. injected with [^{18}F]AlF-NOTA-SC (n=8), [^{177}Lu]Lu-BL02 (n=4) and [^{161}Tb]Tb-BL02 (n=4)

Organ	[18F]AlF-NOTA-SC	[177Lu]Lu-BL02	[161Tb]Tb-BL02
Kidneys	15.62±5.73	11.58±3.45	9.01±3.35
Liver	1.98±1.37	1.93±1.17	2.63±2.40
Spleen	0.81±0.30	0.91±0.22	0.45±0.09
Pancreas	0.53±0.20	0.65±0.22	0.26±0.07
Lungs	1.91±0.89	2.47±0.69	0.97±0.07
Heart	0.64±0.31	0.76±0.25	0.30±0.02
Blood	1.15±0.68	1.54±0.63	0.50±0.05
Bone	2.48±0.98	0.49±0.15	0.55±0.10
Muscle	0.58±0.35	0.69±0.34	0.25±0.10
Brain	0.09±0.05	0.10±0.04	0.02±0.00
Tu-mor_MM.1S	15.42±3.53	14.26±1.99	11.38±2.76

Table S4 SUV values of dissected organs from the *ex vivo* biodistribution study at 7d p.i. injected with [177Lu]Lu-BL02 (n=4).

Organ	[177Lu]Lu-BL02
Kidneys	0.05±0.00
Liver	0.05±0.01
Spleen	0.00±0.00
Pancreas	0.08±0.04
Lungs	0.01±0.01
Heart	0.00±0.00
Blood	0.00±0.01
Bone	0.00±0.00
Muscle	0.01±0.00
Brain	0.00±0.00
Tu-mor_MM.1S	0.11±0.03

Table S5 Percent injected dose (%ID) values of dissected organs from the *ex vivo* biodistribution study at 7d p.i. injected with [^{177}Lu]Lu-BL02 (n=4)

Organ	[^{177}Lu]Lu-BL02
Kidneys	0.08±0.00
Liver	0.29±0.06
Spleen	0.00±0.00
Pancreas	0.03±0.01
Lungs	0.01±0.00
Heart	0.00±0.00
Blood	0.00±0.00
Bone	0.10±0.02
Muscle	0.02±0.01
Brain	0.00±0.00
Tumor_MM.1S	0.40±0.19

Table S6 Percent injected dose per gram of tissue (%ID/g) values of dissected organs from the *ex vivo* biodistribution study at 7d p.i. injected with [^{177}Lu]Lu-BL02 (n=4)

Organ	[^{177}Lu]Lu-BL02
Kidneys	0.24±0.01
Liver	0.23±0.05
Spleen	0.01±0.00
Pancreas	0.40±0.20
Lungs	0.03±0.03
Heart	0.01±0.00
Blood	0.02±0.03
Bone	0.00±0.00
Muscle	0.04±0.01
Brain	0.00±0.00
Tumor_MM.1S	0.52±0.15
